# Evaluation of a Novel Immunoassay for Quantification of C1q for Clinical Diagnostic Use

**DOI:** 10.3389/fimmu.2019.00007

**Published:** 2019-01-25

**Authors:** Kerstin Sandholm, Barbro Persson, Lillemor Skattum, Gösta Eggertsen, Dag Nyman, Iva Gunnarsson, Elisabet Svenungson, Bo Nilsson, Kristina N. Ekdahl

**Affiliations:** ^1^Linnaeus Center of Biomaterials Chemistry, Linnaeus University, Kalmar, Sweden; ^2^Department of Immunology, Genetics and Pathology, Rudbeck Laboratory, Uppsala University, Uppsala, Sweden; ^3^Section of Microbiology, Department of Laboratory Medicine, Immunology and Glycobiology, Lund University, and Clinical Immunology and Transfusion Medicine, Lund, Sweden; ^4^Department of Laboratory Medicine, Karolinska Institutet, Stockholm, Sweden; ^5^Karolinska University Laboratory, Clinical Chemistry, Stockholm, Sweden; ^6^Åland Borrelia Group, Åland Central Hospital, Mariehamn, Finland; ^7^Rheumatology Unit, Department of Medicine, Karolinska Institutet, Karolinska University Hospital, Stockholm, Sweden

**Keywords:** C1q, immunoassays, plasma, CSF, SLE, nephritis

## Abstract

**Objectives:** C1q is a valuable biomarker of disease activity in systemic lupus erythematosus (SLE). The “gold standard” assay, rocket immunoelectrophoresis (RIE), is time-consuming, and thus a shift to soluble immune precipitation techniques such as nephelometry has occurred. However, quantification of C1q with these techniques has been questioned as a result of the antibody binding properties of C1q. In the present work, we have compared results using various techniques (RIE, nephelometry, and ELISA) and have developed and validated a new magnetic bead-based sandwich immunoassay (MBSI).

**Methods:** C1q was quantified by nephelometry and the new sandwich immunoassay in 45 serum samples analyzed using RIE. C1q was also assessed in plasma using RIE and sandwich immunoassay in samples from SLE patients with nephritis (*n* = 69), SLE patients without nephritis (*n* = 310) as classified by BILAG score, and matched controls (*n* = 322). In addition, cerebrospinal fluid (CSF) samples from 31 patients, previously analyzed with ELISA, were also analyzed with the MBSI to test the behavior of this new assay in the lower detection range.

**Results:** We found a strong correlation between the new MBSI, RIE, and ELISA, but not with nephelometry. The MBSI demonstrated lower levels of C1q in SLE patients than in matched controls (*p* < 0.0001), and patients with nephritis had lower levels than patients without nephritis (*p* < 0.01). Similarily, RIE showed significant differences between the patient groups (*p* < 0.0001). An association was also found between the levels of C1q and the SLE disease activity index (SLEDAI). Furthermore, there was good correlation between the values obtained by MBSI and ELISA, in both serum (*r* = 0.960) and CSF (*r* = 0.786), underscoring the ability of both techniques to measure low concentrations of C1q with high accuracy.

**Conclusion:** The sandwich immunoassay correlated well with RIE, but soluble immune precipitation techniques, such as nephelometry, did not appear suitable alternatives, since C1q itself, and possibly anti-C1q antibodies, interfered with the measurements. The new sandwich immunoassay is therefore a good replacement for RIE in monitoring SLE disease activity.

## Introduction

The complement system is involved in many diseases and pathological conditions, including autoimmune disease, infections, cancer, allogeneic and xenogeneic transplantation, and inflammation (1). C1q, the initiator component of the classical complement system, is a powerful effector of the innate immune system and is responsible for pathogen recognition, targeting, and removal (2). The involvement of C1q in apoptotic cell clearance and linkage of its deficiency to the development of lupus is well known (3–6). C1q also has other complement-related and non-complement-related functions and plays a part during pregnancy, wound healing, and aging (7, 8). The involvement of C1q in the pruning and elimination of central nervous system synapses and its requirement for normal brain wiring have recently been discovered (9, 10). C1q has also been demonstrated to act as an external component of the extracellular matrix, favoring tumor growth, and invasion (11).

Systemic lupus erythematosus (SLE) is a systemic disorder in which the formation of immune complexes (ICs) as the result of the generation of autoantibodies is a pivotal mechanism of disease. Therefore, complement activation (consumption) is a common feature during SLE flares and is especially obvious in flares of lupus nephritis. ICs trigger complement activation via the classical pathway, initiated by the binding of the recognition molecule C1q to the immunoglobulins IgG and IgM in the ICs (12). As a consequence of this binding and activation of the complement components of the classical and the terminal pathways, these components are consumed during exacerbations. In addition, activation products such as C3a, C3dg, Bb, and sC5b-9, are generated during flares. By monitoring these markers, the activity of the disease can be followed, and flares can be predicted in many patients (13).

The most commonly used complement activation markers of SLE in routine clinical practice are C4 and C3, which can be analyzed by most clinical laboratories. The specificity and sensitivity of these measures are, however, low and require that earlier results are always available for comparison in order to follow individual patients (13). Furthermore, certain SLE patients have a hereditary lack of C4 resulting from a low number of gene copies encoding C4, which further underscores the conclusion that C4 levels are not an optimal marker of disease in these patients (14, 15).

The first indication that C1q is a useful marker of disease activity in SLE came from Jonsson et al. in 1995 who used rocket immune electrophoresis (RIE) to quantify C1q, a technique which has remained the gold standard for a long time (16). They demonstrated that the predictive value of various parameters for SLE flares was ranked as follows, listed from to highest to lowest predictability: low C1q >high C1-INH/C1r-C1s complexes>low C3 >high C3d >low C4 (14). However, it should be kept in mind that complement analyses generally have rather low sensitivity and specificity, and consequently there are only a limited number of conditions in which serological complement biomarkers can be used as differential markers of disease. On the other hand, such markers are useful for following individual patients, e.g., during SLE flares, if the basal levels are known (13).

Quantification of C1q is today a well-established approach for monitoring SLE disease activity; it can be performed using a number of different techniques: e.g., by RIE (16), nephelometry or turbidimetry (common in clinical routine laboratories), and ELISA. The enthusiasm for C1q measurements varies at different centers, and a suspicion that the various techniques give different results has grown. A possible mechanism (unwanted binding to ICs) that could compromise these measurements reflects the fact that C1q is an IC-binding protein, and most techniques for measurement of C1q are based on IC formation.

Therefore, we decided to compare the various assays used for C1q determination. We found that nephelometry, a fluid-phase immunoprecipitation assay, performed poorly when compared to the original RIE assay and a commercially available ELISA that uses a pair of monoclonal antibodies (mAbs). We then proceeded to use the same pair of mAbs used in the ELISA to develop a magnetic bead sandwich immunoassay (MBSI), which we validated against RIE and ELISA using 45 samples from patients with varying levels of C1q, including several with autoantibodies against C1q. We then validated the MBSI in cerebrospinal fluid (CSF) samples previously analyzed by ELISA. Finally, we measured C1q in a large clinical collection of samples from SLE patients and matched controls by MBSI and compared the results to those obtained with the original RIE. We report here that the MBSI assay was at least as good as RIE with respect to association with the SLE disease activity index (SLEDAI) and flares.

## Materials and Methods

### Complement Standard Serum

Blood was collected from 45 blood group B Rh+ donors at the section for Clinical Immunology and Transfusion Medicine, Uppsala, stored overnight at +4°C, and then centrifuged at +4°C. The resulting serum was pooled, aliquoted, and frozen at −80°C until use, and this pool was referred to as Complement standard serum (17). The C1q concentration in the serum pool was calibrated against C1q-depleted serum (18) spiked with purified C1q, measured by MBSI, and calculated to be 150 mg/L.

### Normal Plasma

EDTA-plasma from 100 healthy blood donors was analyzed by MBSI to obtain a reference interval.

### Clinical Samples (Summarized in Table 1)

The clinical samples analyzed in this study were collected at three different hospitals:

1. Clinical Immunology and Transfusion Medicine, Region Skåne, Lund, Sweden: 85 serum samples from patients with various diagnoses, previously analyzed in the clinical routine laboratory using RIE and selected according to their C1q levels without reference to diagnosis, were anonymized and used for the comparison of the various C1q assays. All samples were stored at −80°C. Forty of the samples were included in an initial methodological comparison (= Group I); the remaining 45, including 5 that were positive for anti-C1q autoantibodies, were used for optimization and validation of the MBSI assay (= Group II).
2. Åland Central Hospital: CSF from 31 patients with suspected neuro borreliosis (stored at −80°C), previously analyzed by ELISA (19) (= Group III), were selected for comparison with MBSI. The study was approved by the Ethics Committee of Åland, 26/5/2005.
3. Clinic of Rheumatology, Karolinska University Hospital Solna, Sweden: All SLE patients, >18 years old, who fulfilled four or more of the 1982 revised American College of Rheumatology (ACR) classification criteria for SLE (*n* = 379) during the inclusion period 2004–2010 were asked to participate; we applied no other exclusion criteria (= Group IV). All consenting participants underwent a structured interview and a physical examination by a rheumatologist (20). Of the participating SLE patients, 69 had current renal involvement at the time of enrolment according to renal British Isles Lupus Assessment Group (BILAG) (A+B+C), whereas the remaining 310 patients had SLE which could be active in other organs than the kidneys or no previous renal involvement (D+E) (21, 22). In the SLE patients, the age at diagnosis and disease duration and manifestations, including autoantibodies, were recorded, and the disease activity index (SLEDAI) was calculated (23, 24). EDTA-plasma samples were drawn after overnight fasting and stored at −80°C. The study was designed to investigate SLE, therefore we chose to include population controls selected from the National Patient Registry, with a diagnosis of SLE as the only exclusion criteria. The controls were matched to the first 322 SLE patients for age, gender and region and were invited via letter to participate. The Local Ethics Committee of the Karolinska University Hospital/Karolinska Institutet, Stockholm, Sweden reviewed the study protocol and approved the study. All participants gave informed written consent to participate, #03-556 (031216).

**Table 1:** Overview of the various clinical materials and analytical techniques used in the present work.

**Clinical material**	**Analyses**						**Outcome**
**Group I** Serum, different diagnoses (*n* = 40)	RIE	Nephelometry (#1 Siemens)	Nephelometry (#2 IMMAGE)	ELISA (mAbs WL02 & DJ01)	ELISA (in- house, pAbs)	MBSI (mAbs WL02 & DJ01)	No correlation nephelometry vs RIE or ELISA mAbs WL02 & DJ01 suitable for MBSI
**Group II** Serum, different diagnoses without (*n* = 40) or with (*n* = 5) anti-C1 q antibodies	RIE	–	Nephelometry (#2 IMMAGE)	–	ELISA (in- house, pAbs)	MBSI (mAbs WL02 & DJ01)	Validation of MBSI (serum/plasma)
**Group III** CSF, different diagnoses (*n* = 31)	–	–	–	–	ELISA (in- house, pAbs)	MBSI (mAbs WL02 & DJ01)	Validation of MBSI (CSF)
**Group IV** EDTA-plasma, SLE (*n* = 379) with/without nephritis (BILAG classification) controls *n* = 322	RIE (not controls)	–	–	–	–	MBSI (mAbs WL02 & DJ01)	MBSI similar to RIE in SLE

### C1q Assays (Table 1)

1. ***RIE***. The RIE assay was an in-house assay performed according to Johnsson et al. (16).
2. ***Nephelometry***. Nephelometry was performed in two ways, either with a BN Pro Spec system (Siemens) using reagents from Siemens (= Nephelometry 1) or using an in-house method with an IMMAGE nephelometer (Beckman Coulter, Bromma, Sweden). The in-house nephelometry assay (= Nephelometry 2) was performed using polyclonal sheep anti-human C1q antibodies from the Binding Site (Birmingham, UK).
3. ***ELISA***. ELISA was performed in two ways: First, a commercially available kit (Hycult, Uden, The Netherlands) was used according to the manufacturer's instructions. This kit uses monoclonal mouse antibodies (mAb) anti-human C1q clone WL02 for capture and anti-human C1q clone DJ01 for detection. Second, an in-house assay, described earlier, using rabbit polyclonal anti-human C1q antibodies for both capture and detection (19).
4. ***Magnetic bead sandwich immunoassay (MBSI)***. This magnetic bead-based assay is a newly developed in-house assay employing the two mAbs used in the commercially available ELISA kit from Hycult. Coupling of 3 μg of monoclonal mouse anti-human C1q clone WL02 (Hycult) to 1.25 × 10^6^ magnetic carboxylated microspheres (Bio-Plex Pro Magnetic COOH Beads, BIO-RAD, Hercules, CA, USA) was performed using the Amine Coupling Kit (BIO-RAD) according to the manufacturer's instructions.

Complement standard serum (150 mg/L) was used as standard and was initially diluted 1:400, and then by two-fold serial dilutions so that the final concentration interval was between 375 and 0.73 μg/L; a well with buffer only was used as background. Coupled beads (2,500/well) were placed in microtiter plates, and samples were diluted 1:5,000. As detection antibody, we used 1.0 μg/mL biotinylated mAb anti-human C1q antibody clone DJ01 (Hycult), followed by streptavidin-R-phycoerythrin (streptavidin-PE, BIO-RAD) diluted 1:100. The final volume of assay buffer per well was 125 μL. The analysis software was set to acquire data using 50 μL from each well, and the amount of beads counted was between 200 and 400. The raw data were acquired with a BioPlex MAGPIX Multiplex Reader (BIO-RAD) and expressed as mean fluorescence intensity (MFI). Standard curves were calculated with a five-parameter regression model, and the C1q concentrations were obtained by interpolation from the standard curve. The development and performance of the MBSI is fully described in the Supporting Information. The general principle for magnetic bead-based assays is described in detail on the manufacturer's home page^1^.

### Statistical Analysis

GraphPad Prism 7.0 (GraphPad Software, San Diego, CA, USA) was used to generate the graphs and regression lines and to calculate the Spearman's non-parametric correlation coefficients. The Mann-Whitney test or ANOVA followed by Kruskal-Wallis analysis were used to compare the C1q concentrations in the three groups: SLE patients with and without nephritis and control patients. The significance level is indicated as ^*^p < 0.05, ^**^p < 0.01, ^***^p < 0.001, and ^****^p < 0.0001.

## Results

### Comparison of the Quantification of C1q by RIE, Nephelometry, and ELISA

**Figure 1:**
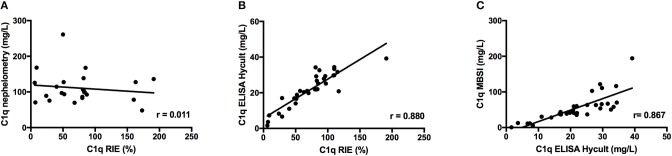
Correlation between C1q concentration in 40 serum samples from patients as determined by RIE and nephelometry **(A)**; RIE and a commercially available ELISA using a pair of mAbs **(B)**; and ELISA and an MBSI constructed using the same pair of mAbs **(C)**.

Initial experiments were performed to quantify C1q by nephelometry and using a commercially available ELISA (employing mAbs) and to compare the results with the results obtained by RIE, which is regarded as the “gold standard” for C1q determination. Results from serum samples selected to have different levels of C1q (Group I) showed negligible correlation between RIE and either nephelometry #1 (*r* = 0.011, Figure 1A) or nephelometry #2 (*r* = 0.563, not shown). In contrast, a significant correlation was found between RIE and ELISA (*r* = 0.880, Figure 1B) and with the in-house ELISA using rabbit polyclonal antibodies (*r* = 0.729, not shown). Based on the correlation between RIE and the commercial ELISA, we decided to use the same pair of mAbs to set up a sandwich immunoassay based on magnetic beads. Given the encouraging results with our new magnetic bead assay (*r* = 0.867, Figure 1C), we then proceeded to develop, optimize, and validate this assay.

### Development of a Magnetic Bead Sandwich Immunoassay (MBSI) for Quantification of C1q

**Figure 2:**
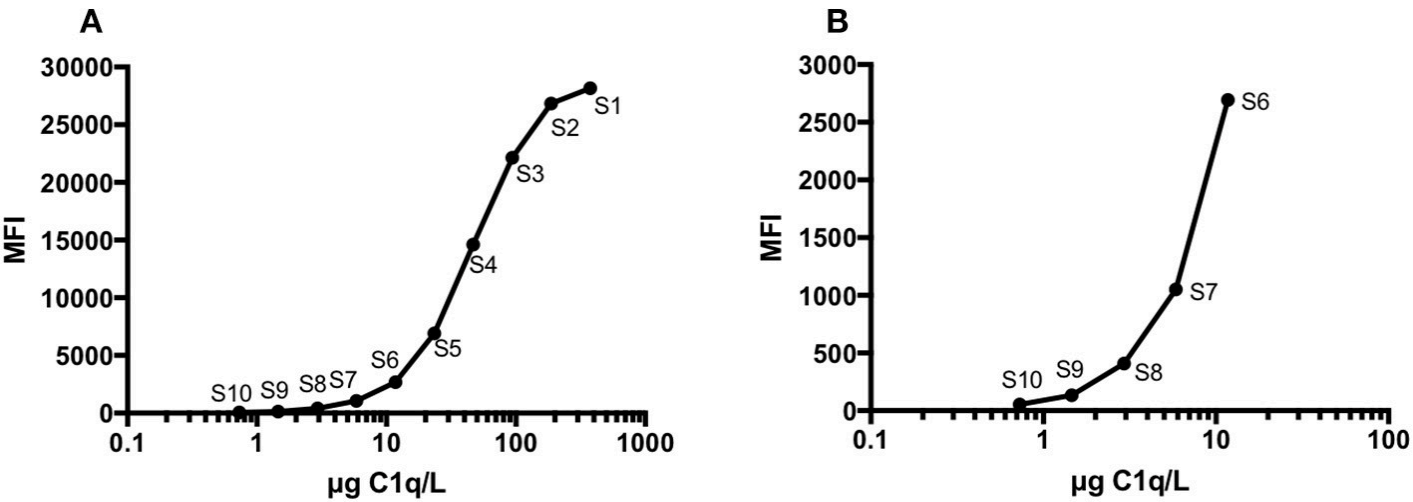
A representative C1q standard curve (S1-S10) obtained with a MAGPIX Multiplex Reader using software BioPlex Manager 6.1 **(A)**. The same standard curve **(B)**, but showing only the five lowest standard concentrations (S6-S10).

The assay was developed for the Mag-Pix platform and potentially allows the assay to be performed together with assays of 40 additional parameters. The measurement interval was 500 times higher than the detection limit and ranged between 375 and 0.73 μg/L, when the plasma samples were diluted 1:5,000 and CSF samples were diluted 1:50 (Figures 2A,B).

The intra-assay coefficient of variation (CV%) was calculated from three plasma samples with different C1q concentrations, which were measured in 10 replicates on the same microtiter plate (Table 2). The intra-assay CV was between 3.2 and 5.4%. Inter-assay CV was calculated from two serum samples with different C1q concentrations measured over the course of 10 days, and the inter-assay CV was between 14.4 and 15.4%.

**Table 2:** Intra- and inter-assay variations in magnetic bead-based sandwich immunoassay C1q assay.

**Intra-assay variation**				**Inter-assay variation**			
***n***	**Mean mg/L**	***SD***	**CV(%)**	***n***	**Mean mg/L**	***SD***	**CV(%)**
10	81.3	2.9	3.5	10	46.7	7.2	15.4
10	132.5	4.2	3.2	10	309.5	44.5	14.4
10	371.0	20.0	5.4				

When serum and EDTA-plasma, collected from the same individual at the same time, are analyzed in parallel, the levels of C1q detected in serum are approximately 5% lower compared to plasma. This is consistent with the fact that C1q binds to activated platelets as reported by our group and others (25, 26). Consequently, there will be a partial depletion of C1q which is entrapped in the clot and removed in the centrifugation process.

### Correlations Between the MBSI, RIE, ELISA, and Nephelometry

**Figure 3:**
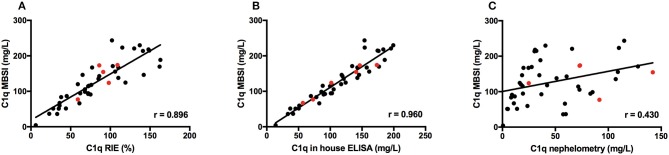
Correlations between C1q concentration in 45 serum samples from patients as determined by RIE and MBSI **(A)**, ELISA and MBSI **(B)**, and nephelometry and MBSI **(C)**. Results from 5 patients with anti-C1q antibodies are indicated with red symbols.

Forty-five serum-samples (Group II) were analyzed with RIE, nephelometry (#2), in-house ELISA, and MBSI in order to evaluate the newly developed MBSI. The C1q concentration ranged from 4.3 to 244 mg/L. A close correlation was found between RIE and the MBSI (r = 0.896; Figure 3A), and between ELISA and the MBSI (*r* = 0.960; Figure 3B). In contrast, no correlation was found between nephelometry and the MBSI (*r* = 0.430; Figure 3C).

### Correlations Between ELISA and the MBSI as Applied to the Determination of C1q in CSF

**Figure 4:**
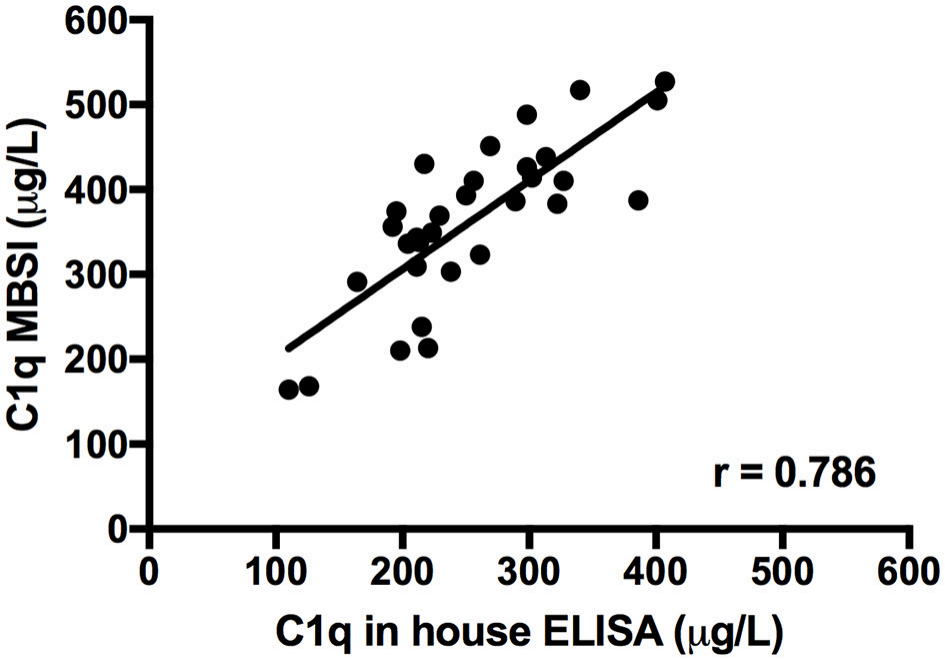
Correlation between C1q levels detected in CSF samples from 31 patients using an in-house ELISA and the MBSI assay.

The concentration of C1q in CSF was measured with both ELISA (19) and MBSI and showed a good correlation between the two methods, *r* = 0.786 (Figure 4).

### Normal Plasma

**Figure 5:**
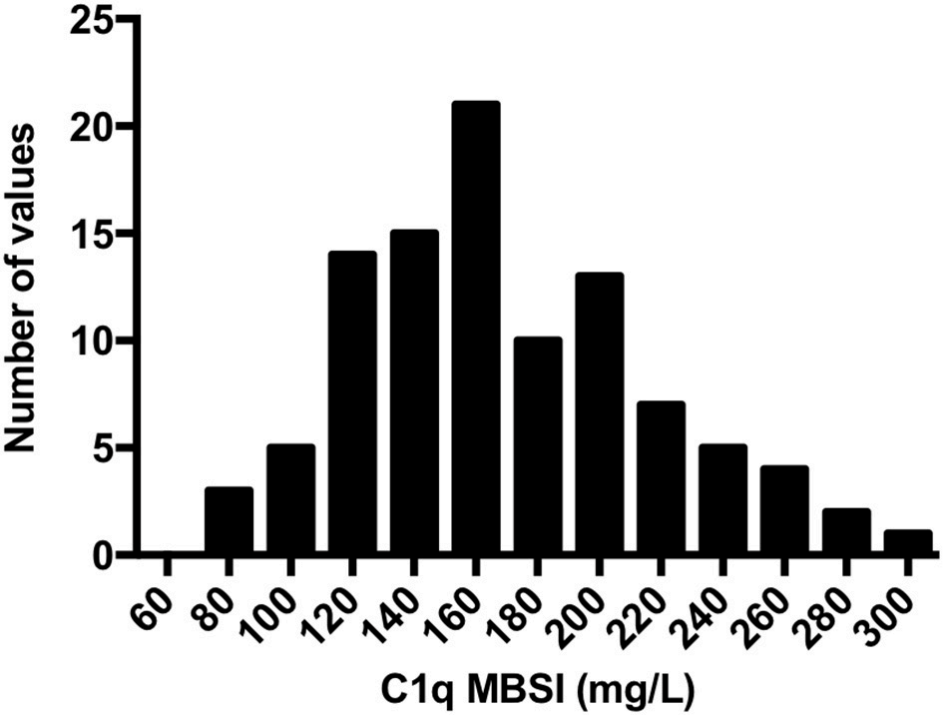
Distribution of C1q concentrations as assessed by MBSI in EDTA-plasma from 100 healthy controls. The reference interval (mean ± 2 *SD*) was calculated to be 170 (mean), with a range of 76–264 mg/L.

The reference interval (mean ± 2 *SD*) was derived from EDTA-plasma from 100 healthy blood donors and was calculated to be 76–264 mg/L (Figure 5).

### C1q in Samples From SLE Patients

**Figure 6:**
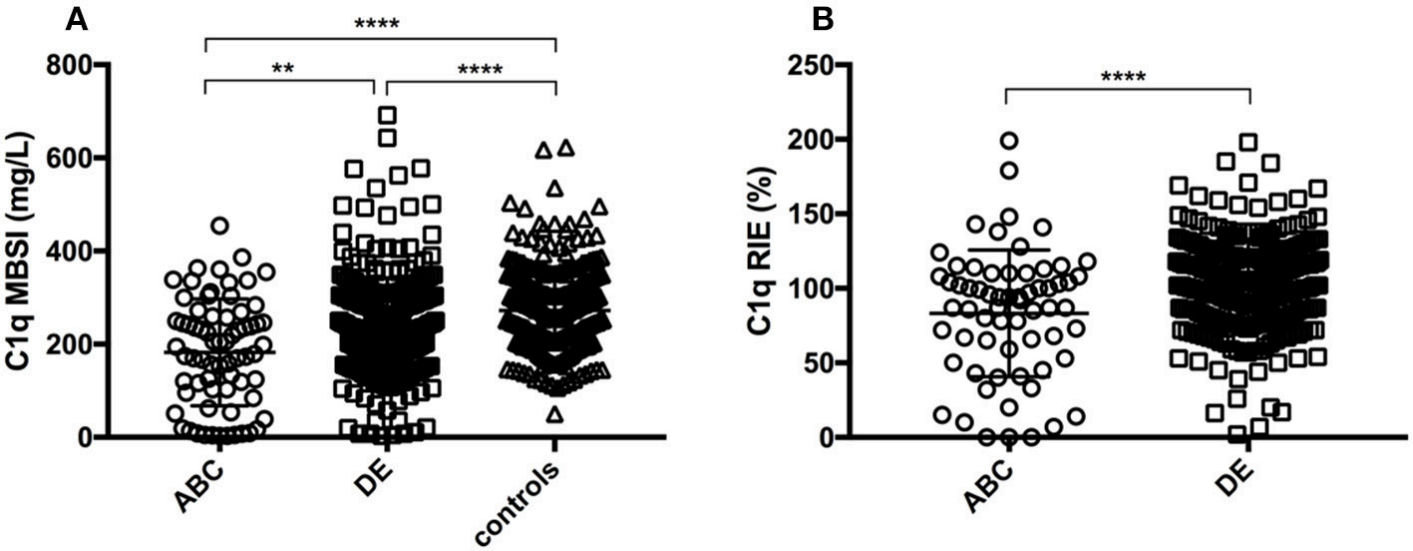
C1q concentrations in EDTA-plasma as measured with MBSI **(A)** and RIE **(B)**. Renal disease activity was assessed by the BILAG index. Current renal activity (A+B+C) and no current renal activity (D+E); controls are patients without SLE. There were significant differences between the SLE groups and controls in the MBSI and between the SLE groups in the RIE. Median values of C1q by MBSI in the various groups were: SLE with nephritis, 194 mg/L; SLE without nephritis, 228 mg/L; and controls, 266 mg/L **(A)**; by RIE: SLE with nephritis, 89.5%, and SLE without nephritis, 105% **(B)**. ^**^p < 0.01; ^****^p < 000.1.

The concentration of C1q in SLE patients with or without nephritis was measured with both the MBSI and RIE. In addition, C1q in matched controls was measured using the MBSI. The levels were significantly lower in the total group of SLE patients (median, 225 mg/L; range, 3.2–691.7 mg/L) than in the controls (median, 266 mg/L; range, 50.5–623 mg/L; *p* ≤ 0.0001) when determined by MBSI. Furthermore, SLE patients with nephritis (median, 194 mg/L; range, 3.2–545 mg/L) had significantly lower levels of C1q than did patients without nephritis (median, 228 mg/L; range, 3.2–692 mg/L; *p* < 0.01), when assessed by MBSI (Figure 6A). The corresponding values for C1q quantified by RIE were 89.5 mg/L (range, 0–199 mg/L) for the patients with renal involvement and 105 mg/L (range, 2–198 mg/L; *p* ≤ 0.0001) for those without renal involvement (Figure 6B).

### Relationship Between the C1q Concentration and SLEDAI

**Figure 7:**
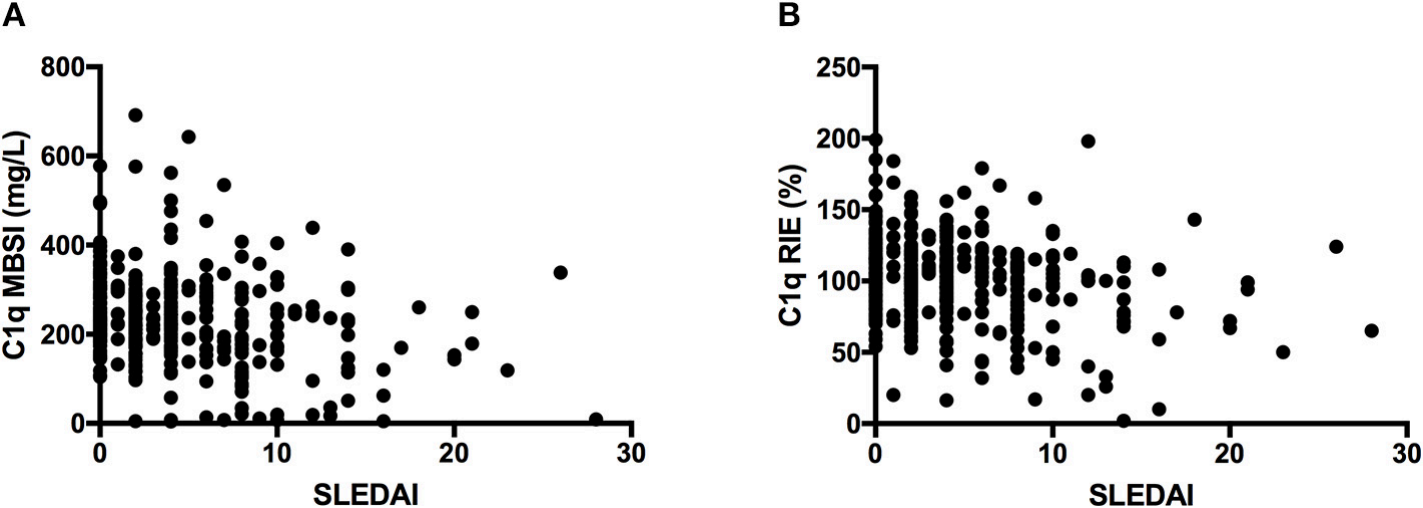
Plots of C1q concentration vs. SLEDAI, as assessed with MBSI **(A)** and RIE **(B)** in the SLE patients, showing an association between the values measured with MBSI but less association with RIE. In the case of the MBSI measurements, the SLE patients with higher SLEDAI had consistently lower C1q values.

A relationship was found between the concentrations of C1q and SLEDAI when the concentration was assessed either by MBSI or by RIE (Figures 7A,B). The association was reflected in the fact that only one patient with a SLEDAI >15 had C1q concentrations above the mean for normal individuals. In contrast, at lower SLEDAI indices, the C1q concentration could be either high or low.

### Lack of a Relationship Between the C1q Concentration and anti-dsDNA

No relationship was found between the concentrations of C1q and anti-dsDNA if the C1q concentration was assessed with either MBSI or RIE (data not shown).

## Discussion

Since the first demonstration that C1q level as assessed using RIE is a useful biomarker for SLE disease activity (16), C1q has been measured by a variety of different assays without any further evaluation of their accuracy or relationship to disease activity (27–29). Here we describe a new assay for quantifying C1q in EDTA-plasma and CSF. The assay is a magnetic bead-based sandwich immunoassay that is a further development of a commercially available ELISA employing the same pair of mAbs.

We were able to establish a strong correlation between ELISA and the new MBSI. The new assay was sensitive, the standard curve had a wider range than either of the ELISAs, and the inter-and intra-assay variations were low. The assay also has the potential to be used in multiplex assays. The new assay showed a high correlation with RIE (*r* = 0.90; *p* ≤ 0.0001), making it suitable to replace this assay, which is more time-consuming to perform. In contrast, nephelometry showed a very poor correlation with RIE, demonstrating that the two assays measure different aspects of C1q levels; the reason for this difference may be related to the biological properties of C1q: C1q has a high affinity for IgG- and IgM-containing ICs under conditions of antibody excess, and since soluble immunoprecipitation assays such as nephelometry and turbidimetry are based on the formation of ICs in antibody excess, it is very likely that C1q further aggregates the ICs, thereby interfering with the assay. In both RIE and in a sandwich immunoassay, the conditions are slightly different with respect to antibody/antigen proportions, in that the proportion is more toward antigen excess, or at least equilibrium. In addition, neither the MBSI nor the RIE was affected by the presence of the anti-C1q antibodies often found in SLE patients, whereas the nephelometry measurements were highly affected by them. This difference also helps explain the poor correlation we saw between RIE and nephelometry.

By applying the new assay to a large group of SLE patients and comparing their results with those for matched healthy controls, we were able to demonstrate that the levels of C1q were significantly lower in the patients. Subdivision of the SLE group into those patients with current renal involvement and those with whose SLE was inactive or without renal involvement demonstrated that the nephritis group had the lowest C1q levels. The SLE group, but not the matched controls, were also assessed by RIE and gave results similar to those obtained with the MBSI.

The data obtained from the MBSI and RIE were also correlated with the SLEDAI indices of the patients. In the case of the MBSI, high SLEDAI indices only seldom coincided with high or normal C1q levels, whereas at low SLEDAI, the C1q levels were more variable. Earlier studies using RIE have shown similar inverse associations between SLEDAI and C1q levels (30, 31).

In addition to the usefulness of monitoring C1q levels in lupus, there are other potential applications for C1q as a marker in pathological disorders, some of which are mentioned here: (1) Increased C1q levels in peripheral blood have been found to be associated with active tuberculosis (TB), as compared to latent TB infection. C1q therefore has potential as a diagnostic marker to discriminate active TB from latent TB infection as well as TB pleurisy from non-TB pleurisy (32). (2) Serum C1q levels are higher in middle-aged and older adults than in younger adults, and they correlate with muscle mass and muscle strength. The C1q level may therefore reflect the loss of muscle mass and strength that occurs with advancing age, and C1q therefore has potential as a novel biomarker of sarcopenia (33). (3) Low levels of C1q have been demonstrated to have a strong correlation to a diagnosis of acquired angioedema (caused by autoantibodies to C1-INH) suggesting that C1q is a useful tool also in this context (34). (4) In bipolar disorders (BD), the serum complement factor levels are significantly reduced in those with chronic BD as compared to first-episode BD patients, perhaps because of overconsumption of the complement system, suggesting that complement factors could serve as indicators of disease severity, neural loss, and cognitive dysfunction (35).

The MBSI can measure C1q when present at low concentrations, and the assay therefore makes it possible to analyze C1q levels in CSF, offering the potential for applications, like the previous described ELISA, measure C1q in suspected neuroinflammatory conditions (19). The blood-brain barrier normally protects the brain from plasma-derived complement and infiltrating immune cells, but complement components can be locally produced in the brain, most often in response to injury or inflammatory signals (36). In the CNS, C1q is involved in the pruning of synapses (9, 10). The local production of C1q in the CNS affects the synaptic plasticity in both normal aging and the course of pathological changes. Apoptotic neurons activate complement pathways, and complement factors C1q and C3 are increased in Alzheimer's disease; also, in schizophrenia patients, complement factors appear to be upregulated (9, 36). CSF levels of C1q and other complement factors are elevated during bacterial meningitis, and the pattern of complement activation differs between patients with pneumococcal and those with meningococcal meningitis (37). Also, in non-HIV cryptococcal meningitis, the initiators of the three complement pathways (C1q, factor B, and MBL) and other complement factors are increased (38).

In summary, we have developed a novel assay for quantifying C1q in EDTA-plasma of SLE patients as well as in CSF, and the opportunity exists for more applications of the assay for C1q as a marker in other pathological disorders. This assay is convenient and easy to use and can potentially be applied in a multiplex setting. In contrast to soluble immunoprecipitation assays, such as nephelometry and turbidimetry, it correlates well with the gold standard assay, RIE, and was evaluated and shown to give a clear link to nephritis and SLEDAI in SLE patients.

## Author Contributions

KS, BN, and KE designed the research project. KS, LS, GE, IG, and ES performed the research. KS, BP, BN, and KE wrote the manuscript with editorial help from LS, DN, IG, and ES. All authors approved the final manuscript.

### Conflict of Interest Statement

The authors declare that the research was conducted in the absence of any commercial or financial relationships that could be construed as a potential conflict of interest.

## Footnotes

^1^https://www.luminexcorp.com/xmap-technology
