# Supplementary material for: Evaluation of a Novel Immunoassay for Quantification of C1q for Clinical Diagnostic Use
Source: Front Immunol. 2019 Jan 25;10:7. doi: 10.3389/fimmu.2019.00007 (PMC6357986; doi:10.3389/fimmu.2019.00007)
Supplement: Supplementary file 1 [file Data_Sheet_1.docx]

**SUPPORTING INFORMATION**

**Magnetic bead-based sandwich immunoassay (MBSI)**

***A. Antibody coupling to microspheres.*** Coupling of antibodies to magnetic carboxylated microspheres (Bio-Plex Pro Magnetic COOH Beads, BIO-RAD, Hercules, CA, USA) was performed using the Amine Coupling Kit (BIO-RAD) according to the manufacturer´s instructions. The antibody was a monoclonal mouse anti-human C1q clone WL02 (Hycult, Uden, The Netherlands) in phosphate-buffered saline (PBS; 10 mM sodium phosphate buffer, pH 7.4, with 0.145 M NaCl). Five different concentrations of anti-C1q antibody (1.5 µg, 3.0 µg, 6.0 µg, 9.0 µg, and 12 µg) were coupled to portions of beads (1.25x10^6^). To confirm the antibody coupling and to find the optimal concentration of capture antibody, biotinylated polyclonal rabbit anti-mouse immunoglobulins (Dako, Glostrup, Denmark) was used in eight concentrations from 5.7 to 0 µg/ml. The biotinylated antibody was used with streptavidin-R-phycoerythrin (streptavidin-PE) BIO-RAD), diluted 1:100.

The optimal coupled antibody concentration was found to be 3 µg/1.25x10^6^ beads. Coupled beads that had been stored in the dark at 4°C for 12 months were tested again and displayed the same MFI values.

***B. Detection antibody.*** The optimal concentration of detection antibody was tested by titration with biotinylated monoclonal mouse anti-human C1q antibody clone DJ01 (Hycult) at three concentrations (0.5, 1.0 and 2.0 µg/mL) and, 1.0 µg/ml was chosen for further use.

***C. Standard curve.*** For the standard curve, Complement standard serum (150 mg/L) was diluted 1:400 and then in a two-fold serial dilution to yield a concentration range of 375 µg/L to 0.73 µg/L. A well with assay buffer alone was used as background.

***D. Procedure.*** Standards, controls, samples, detecting antibody, and streptavidin-PE were all diluted in assay buffer (PBS supplemented with 0.1 % bovine serum albumin [BSA; Sigma, St. Louis, MO, USA] and 0.02% Tween 20 [Sigma]). Before use, the assay buffer was filtered with a sterile syringe with a 0.2-µm cellulose acetate membrane filter (VWR, Stockholm, Sweden).

A handheld magnetic washer (BIO-RAD) was used to separate the coupled microsphere after each incubation and wash cycle. PBS with 0.05% Tween 20 (Sigma) was used as a washing buffer. All incubations were performed at room temperature, for 30 min, in dark and under rotation at 850 rpm, using a Titramax 1000 (Heidolph Instruments GmbH, Schwabach, Germany). The assay was performed in 96-well flat-bottom microtiter plates (Bio-Plex Pro, BIO-RAD), and antibody-coupled beads were used at a concentration of 2500 beads per well. All standards, controls, and samples were tested in duplicate. Fifty µL of Complement standard serum, controls, or sample, each diluted in assay buffer, was added to each well, and the plate was incubated for 30 min as detailed above. After three washes with 200 µL washing buffer/well, 50 µL biotinylated anti-C1q antibody/well was added, and the plate was incubated again. The beads were washed as described previously, and 50 µL of streptavidin-PE/well, diluted 1:100 in assay buffer, was added. After another 30-min incubation and and three washes, the beads were resuspended in 125 µL assay buffer/well, and the plate was rotated for 10 secs at 850 rpm before the mean fluorescence in intensity (MFI) was measured in a BioPlex MAGPIX Multiplex Reader (BIO-RAD) using Bio-Plex manager MP Software and Bio-Plex Manager 6.1 (BIO-RAD) to calculate the results (**Figure 1**). In the final assay, the samples were diluted in assay buffer (1:5000); 2500 anti-C1q-coupled beads/well were added, and detected was performed using biotinylated anti-C1q (1 µg/mL) followed by streptavidin-PE (1:100).
